# Supplementary figures and images for: Simultaneous Isolation and Purification of Transferrin and Immunoglobulin G from Human Serum—A New Biotech Solution
Source: Molecules. 2025 Feb 21;30(5):993. doi: 10.3390/molecules30050993 (PMC11901732; doi:10.3390/molecules30050993)

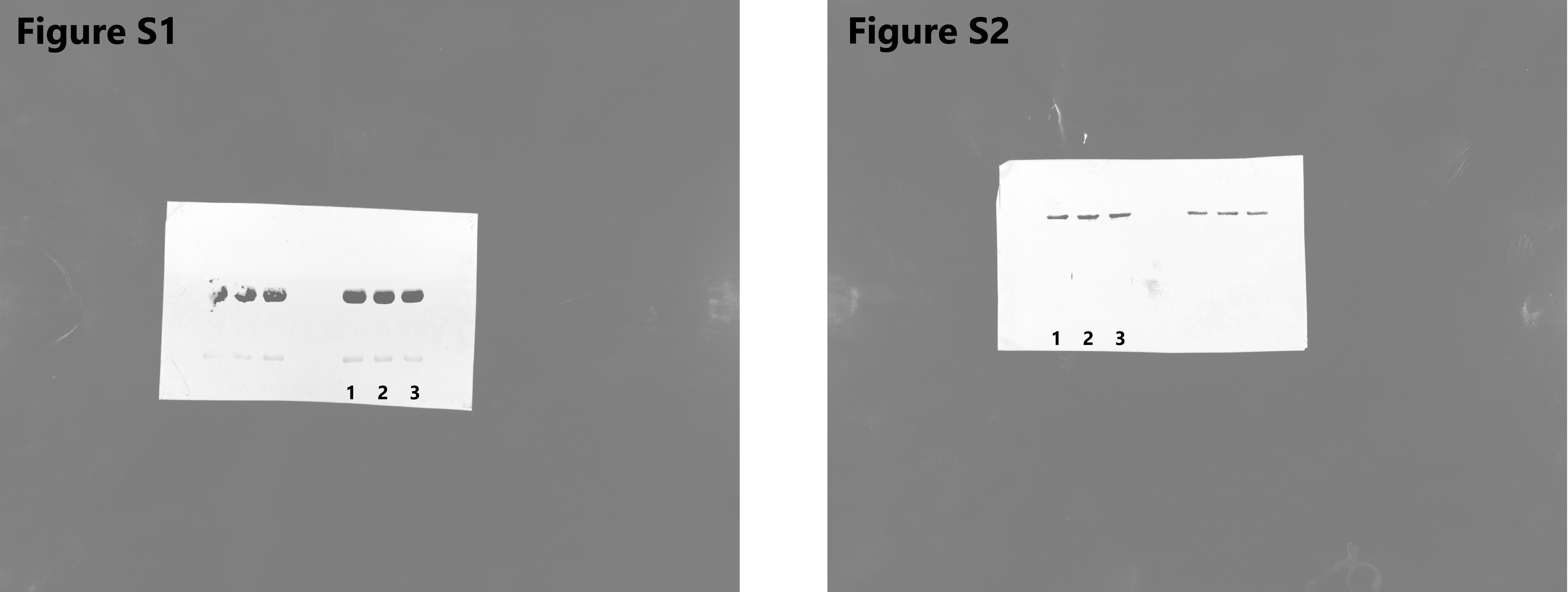

Supplement: Supplementary file 1 [file molecules-30-00993-s001.zip › molecules-3445204-supplementary.tiff]
